# Predictors of adverse perinatal and maternal outcomes of instrumental vaginal delivery at a tertiary setting in Ethiopia: A cross‐sectional study

**DOI:** 10.1002/puh2.41

**Published:** 2022-12-07

**Authors:** Abraham Fessehaye Sium, Wondimu Gudu, Don Eliseo III Lucero‐Prisno, Aida Tilahun

**Affiliations:** ^1^ St. Paul's Hospital Millennium Medical College, Department of Obstetrics and Gynecology Addis Ababa Ethiopia; ^2^ Department of Global Health and Development, London School of Hygiene and Tropical Medicine London UK; ^3^ Faculty of Management and Development Studies, University of the Philippines (Open University), Los Baños Laguna Philippines

**Keywords:** forceps delivery, instrumental delivery, perinatal complications, vacuum delivery

## Abstract

**Objective:**

To determine the rate of instrumental vaginal delivery (IVD) and the predictors of adverse maternal and fetal outcomes associated with it in an Ethiopian setting.

**Methods:**

A cross‐sectional study was conducted from October 1, 2018, to January 31, 2019, at St. Paul's Hospital Millennium Medical College (SPHMMC) (Addis Ababa, Ethiopia). Data on obstetric characteristics, perinatal and maternal outcomes of women who delivered through IVD were collected prospectively, using a structured questionnaire. Data were analyzed using SPSS version 22 and descriptive analysis was applied to analyze baseline characteristics. Multivariable logistic regression model was fitted to predict the association between short‐term complications of IVD and their determinants. Odds ratio, 95% CI, and *p*‐value < 0.05 were used to present significance of study findings.

**Results:**

There were 3165 deliveries during the study period, out of which 241 (7.6%) were instrumental vaginal deliveries. Sequential use of instrumental delivery (AOR = 4.82 [95% CI = 2.10–27.29] and AOR = 6.43 [95% CI = 1.19–34.73], for maternal and fetal complications, respectively) was associated with increased both maternal and fetal complications. Three number of pulls during the extraction was associated with increased fetal complications (AOR = 1.19 [95% CI = 1.05–1.67]).

**Conclusion:**

The rate of instrumental delivery rate in our setting is high with sequential use of instrumental delivery found to be associated with increased adverse maternal and fetal outcomes while three number of pulls were associated with increased fetal adverse outcomes.

## INTRODUCTION

The caesarean section (CS) rate continues to increase across high‐income, middle‐income, and low‐income countries. Latest data (2010–2018) from 154 countries covering 94.5% of live births shows that 21.1% of women gave birth by CS worldwide, averages ranging from 5% in sub‐Saharan Africa to 42.8% in Latin America and the Caribbean. [1] Appropriate use of instrumental delivery using vacuum or forceps is an alternative procedure and it reduces the risks associated with CS and the costs of obstetric care [2].

Instrumental delivery rate varies greatly between settings and the ideal rate is not known. One in 10 deliveries in the developed world is instrumental delivery [3, 4]. The lowest rates have been reported from south Asia and Sub‐Saharan Africa. A survey done in nine Asian countries by the World Health Organization (WHO) found prevalence of 3.2% . [5] Rates of under 1% have been reported from sub‐Saharan Africa [6]. However national reports show that in Sub‐Saharan Africa, the rate of instrumental delivery is as high as 10.3% in Southwest Ethiopia [7], and as low as 2.4% in Uganda [8] and 2.3% in Cameroon [9].

Although instrumental vaginal delivery (IVD) decreases maternal risk of cesarean delivery, it is associated with serious fetal and maternal complications. Fetal complications include facial or scalp lacerations, skull fracture and/or intracranial hemorrhage, facial nerve palsy, compression injury to cornea, retinal hemorrhage, subaponeurotic/subgaleal hemorrhage, cervical spine injury, and hyperbilirubinemia (from breakdown of hematoma) [10]. Cervical lacerations, vaginal lacerations and/or hematomas, and third‐ and fourth‐degree perineal tears are the maternal complications associated with IVD [11, 12, 13, 14]. This study aimed at determining the rate of adverse maternal and fetal outcomes of IVD and its predictors in an Ethiopian tertiary setting.

## METHODS

### Study design, area, and period

This is a cross‐sectional study conducted at St. Paul's Hospital Millennium Medical College (SPHMMC) which is a leading tertiary hospital in Ethiopia and located in the capital Addis Ababa, with various specialty and sub‐specialty level health care and training programs. The department of obstetrics and gynecology at the college is one of the biggest maternity service centers in Ethiopia, with 11,500 deliveries on average being attended at its obstetric unit every year. Instrumental vaginal delivery (IVD) is a common practice at the obstetric unit and most of these deliveries are attended by post‐graduate Obstetrics and Gynecology residents. The primary outcome of this study was to determine short‐term maternal and fetal complications of instrumental delivery and its predictors. The study population were all pregnant women who had instrumental delivery at SPHMMC during the study period (from October 1, 2018, to January 31, 2019). Exclusion criteria were mothers who had a trial of IVD elsewhere, before referral to SPHMMC and those with lethal congenital fetal anomalies.

### Sample size and sampling procedure

The sample size was calculated using single population proportion formula taking a *p*‐value of 0.83, which is the incidence of the most common fetal complication after IVD taken from a previous study done in Tigray (Northern Ethiopia) [15]. Using the power of 80%, adding a marginal error of 5%, and a 10% non‐response rate, the estimated sample size was 238. Non‐random sampling method was used to recruit study subjects.

### Data collection procedures

Data were collected prospectively using a structured questionnaire prepared in English and by three data collectors under one supervisor. The questionnaire which included information on socio‐demographic data, obstetric characteristics, consent‐obtaining process, and perinatal outcomes, was filled out immediately after delivery by the data collectors. The decision whether to proceed with cesarean section or sequential use of instrumental delivery after a failed instrumental delivery was care provider dependent (no uniform protocol was followed). Questionnaire pretesting was conducted in 5% of clients before actual data collection proceeded. A 3 days of training on data collection, research objectives, and confidentiality of information was provided to data collectors and the supervisor by principal investigator. Ethical clearance was obtained from the SPHMMC Institutional Review Board (IRB). Written informed consent was obtained from study subjects.

### Data processing and analysis

Data was entered to EPI info version 3.1 and exported later to SPSS version 22 for analysis. Descriptive analysis was used to analyze baseline characteristics. Distribution of variables was expressed in frequencies and percentages. Variables with a *p‐value* < 0.25 on bivariate analysis were entered into multivariable analysis. Multivariable logistic regression model was fitted to predict the association between short‐term complications of IVD and its determinants. Odds ratio, 95% CI, and *p*‐value < 0.05 were used to present significance of study findings.

## RESULTS

There were 3165 deliveries at SPHMMC during the study period, out of which 241 (7.6%) were instrumental vaginal deliveries. A total of 238 instrumental deliveries were included in the final analysis. As demonstrated in Table 1, majority of the participants were primigravida (63%) and belonged to age range of 20–34 (87.4%). Similarly, 204/238 (85.7%) had a normal birth weight (2500–3999 g). When it comes to gestational age distribution, 87.5% the cases were term pregnancies (GA = 37–41 ^+^ ^6^ weeks). Vacuum delivery was more commonly practiced than forceps (65.1% vs. 34.9%) and low type of instrumental delivery was observed more common than outlet (83.6% vs. 16.4%). Prolonged second stage was the most common indication for instrumental delivery represented in 45.4% (108/238).

**TABLE 1:** Baseline characteristics of instrumental delivery cases (*n* = 238)

Variable	Frequency	Percentage
**Age**		
< = 19	19	8.0
20–34	208	87.4
35–49	11	4.6
**Parity**		
I	152	63.9
II–V	80	33.6
>V	6	2.5
**Birth weight**		
<2500 g	27	11.4
2500–3999 g	204	85.7
≥4000 g	7	2.9
**Gestational age**		
34–36 ^+^ ^6^ week	19	7.9
37‐41 ^+^ ^6^ week	208	87.5
> = 42 week	11	4.6
**Station of the fetus**		
Low	199	83.6
Outlet	39	16.4
**Type of instrument used**		
**Vacuum**	155	65.1
Metallic	42	26.9
Plastic	113	73.1
**Forceps**	83	34.9
**Episiotomy**		
Yes	177	74.4
No	61	25.6
**Indication for IVD**		
Prolonged SSOL	108	45.4
Shorten SSOL	17	7.1
NRFHRP	113	47.5

Abbreviation: IVD, instrumental vaginal delivery; NRFHRP, non‐reassuring fetal heart rate pattern; SSOL, second stage of labor.

**FIGURE 1:**
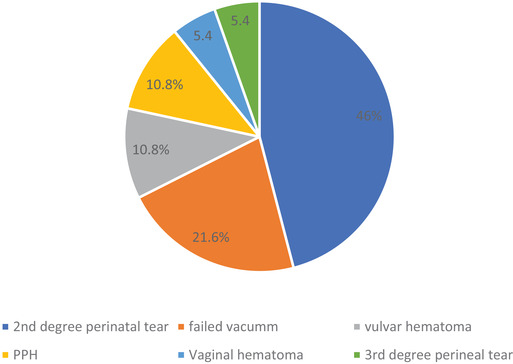
Maternal complications of instrumental vaginal delivery (IVD) (*n* = 37)

In this study, 37out of 238 (15.5%) mothers developed a maternal complication. As indicated in Figure 1, 17 (46%) of the mothers had second‐degree perineal tears, four (10.8%) mothers had post‐partum hemorrhage (PPH) and another 2 (5.4%) had third‐degree perineal tears.

**FIGURE 2:**
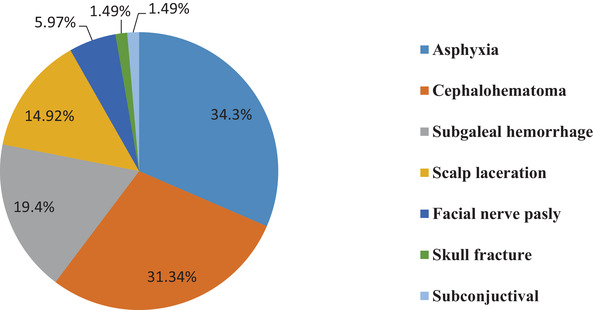
Fetal complications among fetuses delivered via instrumental vaginal delivery (*n* = 67)

From the total instrumental deliveries, 67 (28.15%) of them had fetal complication (Figure 2). Out of this, 23 (34.3%) had birth asphyxia and 21 (31.3%) had cephalhematoma. There were 18 (7.5%) observations of perinatal death.

On multivariate analysis (Tables 2 and 3), sequential use of instrumental delivery (AOR = 4.82 [95% CI = 2.10–27.29] and AOR = 6.43 [95% CI = 1.19–34.73], for maternal and fetal complications, respectively) was associated with increased maternal and fetal complications. Three number of pulls during extraction was associated with increased fetal complications (AOR = 1.19 [95% CI = 1.05–1.67]).

**TABLE 2:** Factors associated with fetal complication in IVD cases

	**Fetal complication**		
Variable	Yes *n*(%)	No *n*(%)	AOR (95% CI)
**Birth weight**			
< 2500 g	13 (19.40)	14 (8.19)	1.38 (0.19–10.17)
2500–3999 g	49 (73.13)	155 (90.64)	1
> = 4000 g	5 (7.46)	2 (1.17)	6.12 (0.04–18.95)
**Gestational age**			
34‐36 ^+^ ^6^ week	12 (17.91)	7 (4.09)	4.65 (0.80–26.98)
37–41 ^+^ ^6^ week	48 (71.64)	160 (93.57)	1
> = 42 week	7 (10.45)	4 (2.34)	0.81 (0.05–14.13)
**Parity**			
P‐I	44 (65.67)	108 (63.16)	0.52 (0.34–13.56)
P‐II‐V	20 (29.85)	60 (35.09)	1
P → V	3 (4.48)	3 (1.75)	0.28 (0.15–12.95)
**Type of IVD**			
**Vacuum**	34 (50.75)	121 (70.76)	1
**Forceps**	33 (49.25)	50 (29.4)	0.43 (0.13–1.46)
**Station of the fetus**			
Low	48 (71.64)	151 (88.3)	4.04 (0.65–13.03)
Out let	19 (28.35)	20 (11.69)	1
**No. of pulls**			
1	16 (23.88)	71 (41.52)	1
2	38 (56.72)	93 (54.39)	1.06 (1.01–1.59)
3	13 (19.40)	7 (4.09)	1.19 (1.05–1.67)
**Sequential use of IVD**			
Yes	10 (14.93)	9 (5.26)	6.43 (1.19–34.73)
No	57 (85.07)	162 (94.74)	1

Abbreviations: AOR, adjusted odds ratio; CI, confidence interval; IVD, instrumental vaginal delivery; No., number; P, parity.

**TABLE 3:** Factors associated with maternal complications

	**Maternal complication**			
**Variable**	**Yes** ***n* (%)**	**No** ***n* (%)**	**COR (95% CI)**	**AOR (95% CI)**
**Parity**				
P‐I	23 (62.16)	129 (64.18)	0.92 (0.43–1.92)	0.82
P‐ II‐V	13 (35.14)	67 (33.33)	1	1
P → V	1 (2.70)	5 (2.49)	0.89 (0.09–7.98)	0.92
**Birth weight**				
< 2500 g	8 (21.62)	19 (9.45)	3.15 (1.25–7.99)*	2.79 (0.32–24.62)
2500–3999 g	24 (64.86)	180 (89.55)	1	1
> = 4000 g	5 (13.51)	2 (1.00)	0.17 (0.03–1.05)	1.51 (0.14–16.17)
**Station of the fetus**				
Low	30 (81.08)	169 (84.07)	0.56 (0.26–1.19)	2.04 (0.29–14.03)
Outlet	7 (18.91)	32 (15.92)	1	1
**Type of instrument used**				
Vacuum	19 (51.35)	136 (67.66)	1	1
Forceps	18 (48.65)	65 (32.34)	0.50 (0.25–1.03)*	1.39 (0.29–6.63)
**Episiotomy**				
Yes	26 (70.27)	151 (75.12)	1	1
No	11 (29.73)	50 (24.88)	0.78 (0.36–1.69)	2.97 (0.27–32.45)
**Sequential use of ID**				
Yes	7 (18.92)	12 (5.97)	3.67 (1.34–10.07)	4.82 (2.10–27.29)
No	30 (81.08)	189 (94.03)	1	1
**No. of pulls**				
3	10 (27.03)	10 (4.98)	0.10 (0.03–0.316)	0.12 (0.01–1.57)
2	19 (51.35)	112 (55.72)	0.59 (0.25–1.43)	0.49 (0.12–2.27)
1	8 (21.62)	79 (39.30)	1	1

Abbreviations: AOR, adjusted odds ratio; CI, confidence interval; COR, crude odds ratio; ID, instrumental delivery; P, parity.

## DISCUSSION

In the present study, the rate of instrumental delivery was found to be 7.6% with maternal and fetal complications associated with instrumental delivery being 15.6% and 28.4%, respectively. On further analysis, sequential use of instrumental delivery was associated with both maternal and fetal adverse outcomes, whereas three pulls during extraction were associated with an increased risk of fetal complications.

The rate of instrumental delivery found in our study is lower than 10.3% reported from Southwest Ethiopia [7], but higher than a report of 2.3% from Cameroon [9], 4.9% from Lagos (Nigeria) [16], and 2.4% from Uganda [8]. According to the literature, maternal complication due to instrumental delivery can range from laceration of vagina and perineum to major complications such as severe hemorrhage, bladder injury, and pelvic muscle injury [15]. In this study, the rate of maternal complication was 15.5%, which is higher than the proportion of maternal complication related to instrumental delivery reported from Northwest Ethiopia [17]. Second‐degree perineal tear was the commonest maternal complication in our study, while PPH was observed in 4 (10.8%) patients.

Consistent with findings of previous studies, sequential use of instrumental delivery was found to be a predictor of adverse maternal and fetal outcomes in the present study (AOR = 4.82 [95% CI = 2.10–27.29] and AOR = 6.43 [95% CI = 1.19–34.73], respectively). Gardella G et al. and Demissie K et al reported increased incidence of adverse maternal and neonatal outcomes associated with the use of sequential instrumental delivery [18, 19]. Another large study, in which 2628 instrumental deliveries were retrospectively reviewed concluded that sequential use of instrumental delivery carries a significantly higher fetal complication [20]. Likewise, Morphy et al. found that the use of multiple instruments was associated with increased neonatal birth trauma injury [21].

Strengths of this study are prospective nature of the study with ideal study design, and analysis of various factors that contribute to increased adverse maternal and fetal outcomes associated with instrumental delivery. Among the main limitation of this study are data collection over a short period of time (4 months), and failure to analyze the perinatal deaths by direct causes of death and determine whether there was a direct association between instrumental delivery and these perinatal deaths. The other limitation is lack of random sampling technique during recruitment of study subjects, which could be a potential source bias.

## CONCLUSION

The rate of instrumental delivery in our setting is high with sequential use of instrumental delivery found to be associated with increased adverse maternal and fetal outcomes while three number of pulls was associated with increased fetal adverse outcomes. These findings highlight the importance of the willingness to abandon the procedure if the instrument being applied fails or sounds inappropriate, instead of shifting to sequential use of instrumental delivery or repeated pulls with the aim of achieving extraction through forceful traction.

## AUTHOR CONTRIBUTIONS

AT contributed conceptualization of the project and the framework for data collection. AT and WG contributed data analysis. AFS and WG contributed data interpretation. AFS, WG, and DEL contributed manuscript write‐up. All authors approved the final manuscript.

## CONFLICT OF INTEREST

The authors declares that they have no financial or non‐financial competing interests. AFS is an Editorial Board member of Public Health Challenges and co‐author of this article. DEL is editor in chief of Public Health Challenges and co‐author of this article. They were excluded from editorial decision‐making related to the acceptance of this article for publication in the journal.

## ETHICS STATEMENT

Ethical clearance & permission letter was obtained from the Institutional Review Board (IRB) of St. Paul's hospital millennium Medical College (SPHMMC). A written informed consent was obtained from each study participant study.

## Data Availability

The data that support the findings of this study are available on request from the corresponding author.
